# Mechanistic Insights into the Abiotic Stress Adaptation of *Salix* Species: A Comprehensive Review of Physiological, Molecular, and Sex-Dimorphic Responses

**DOI:** 10.3390/cimb47090767

**Published:** 2025-09-17

**Authors:** Pengcheng Sun, Fangjing Fan, Yinggao Liu, Fuyuan Zhu

**Affiliations:** 1 National Key Laboratory for the Development and Utilization of Forest Food Resources, The Southern Modern Forestry Collaborative Innovation Center, State Key Laboratory of Tree Genetics and Breeding, Key Laboratory of State Forestry and Grassland Administration on Subtropical Forest Biodiversity Conservation, College of Life Sciences, Nanjing Forestry University, Nanjing 210037, China; pcsun@njfu.edu.cn (P.S.); fanfangjing@njfu.edu.cn (F.F.); liuyg@sdau.edu.cn (Y.L.); 2 State Key Laboratory of Crop Biology, College of Life Sciences, Shandong Agricultural University, Tai’an 271000, China; 3 State Key Laboratory of Desert and Oasis Ecology, Xinjiang Institute of Ecology and Geography, Chinese Academy of Sciences, Urumqi 830011, China

**Keywords:** *Salix* spp., abiotic stress, transcription factors, stress-responsive genes

## Abstract

As key species for ecological restoration, *Salix* spp. thrive in harsh environments, including high-altitude regions, arid zones, and saline–alkaline soils, demonstrating remarkable resilience to abiotic stressors. Recent advances in genomics, transcriptomics, and functional gene research have shed light on the mechanisms underlying *Salix* species’ responses to drought, salinity, heavy metals, and low-temperature stresses. This review systematically synthesizes the physiological, biochemical, and molecular adaptations of *Salix* to abiotic stress, with a particular focus on underexplored areas such as sex-dimorphic regulation and integrated hormone-ROS signaling pathways. We emphasize the dynamic interplay between transcription factors, hormonal crosstalk, and ROS signaling that underpins the stress response, highlighting sex-specific variations that modulate adaptive capacity. Moreover, we discuss the synergistic roles of exogenous additives and rhizosphere microorganisms in enhancing stress resistance. This comprehensive analysis provides critical insights for breeding stress-resilient *Salix* cultivars and for future research into stress adaptation mechanisms in woody plants.

## 1. Introduction

Willows (*Salix* spp.), a widely distributed genus of woody plants, are known for their remarkable adaptability to diverse and often harsh environments [1]. Due to their rapid growth, robust root systems, and high tolerance to abiotic stresses, willows have been widely utilized in ecological restoration, phytoremediation, and afforestation projects in vulnerable habitats such as riverbanks, saline–alkaline lands, and high-altitude plateaus [2].

In recent years, increasing attention has been paid to the mechanisms by which willows respond to abiotic stresses such as salinity, drought, and heavy metal toxicity. Studies have shown that willows employ a variety of strategies, including morphological and physiological adaptations, activation of antioxidant defenses, and osmotic regulation [3]. The application of exogenous substances such as lysine, calcium, silicon, acetic acid, and spermidine has been shown to mitigate stress-induced damage by enhancing antioxidant enzyme activities, modulating hormonal signaling, and improving nutrient uptake [4,5].

At the molecular level, transcription factors (TFs) and associated regulatory networks play a critical role in stress perception and signal transduction. TF families such as Trihelix, WRKY, MYB, HD-Zip, NAC, AP2/ERF, and Hsf have been identified in willows, with increasing evidence supporting their roles in stress adaptation [6,7].

Despite extensive studies on model plants, knowledge of the molecular and physiological mechanisms underlying abiotic stress responses in *Salix* species remains fragmented. While previous work has identified stress-induced physiological changes and candidate genes, an integrated understanding of hormone signaling, transcriptional regulation, and sex-specific adaptation in *Salix* is still lacking. Moreover, how *Salix* species coordinate responses to different abiotic stressors—either independently or in combination—has not been systematically reviewed.

Therefore, this review aims to provide a comprehensive synthesis of the current knowledge on *Salix* responses to abiotic stresses, including drought, salinity, temperature extremes, heavy metals, and UV radiation. Particular emphasis is placed on transcriptional regulation, redox homeostasis, hormone signaling, and the interplay between stress responses and plant sex. We also highlight recent genomic and transcriptomic advances, compare interspecific and sex-dimorphic responses, and propose future directions to guide stress-resilient breeding and functional studies in *Salix*.

### Search Strategy

We performed a bibliometric analysis using the Web of Science. The database used was the Web of Science Core Collection, and the search keywords included “Salix”, “Abiotic Stresses”, “Cold”, “Heat”, “Drought”, “Heavy Metal”, “Salt”, “Strong Light”, “Waterlogging”, and “Mechanical Damage”. For the construction of the network map of the bibliometric analysis, references from the past five years (2020–2025) were selected. For the heatmap of the bibliometric analysis, references from the past ten years (2016–2025) were selected. To generate the network map, we exported the relevant Excel files and used Pajek (version 5.19) and Bibexcel (version 2016-02-20) for analysis. First, the “Keywords Plus” column was selected from the exported Excel file. Using Bibexcel, keyword frequency statistics were performed, and keywords with frequencies below six were removed before calculating keyword co-occurrence frequencies. Bibexcel was then used to generate the co-occurrence matrix, which produced the .net file for visualizing the network map of the bibliometric analysis. The .net and .vet files generated by Bibexcel were imported into Pajek for data visualization, centrality calculations, and layout adjustments. Finally, VOSviewer (version 1.6.19) was used to refine the visualization.

**Figure 1:**
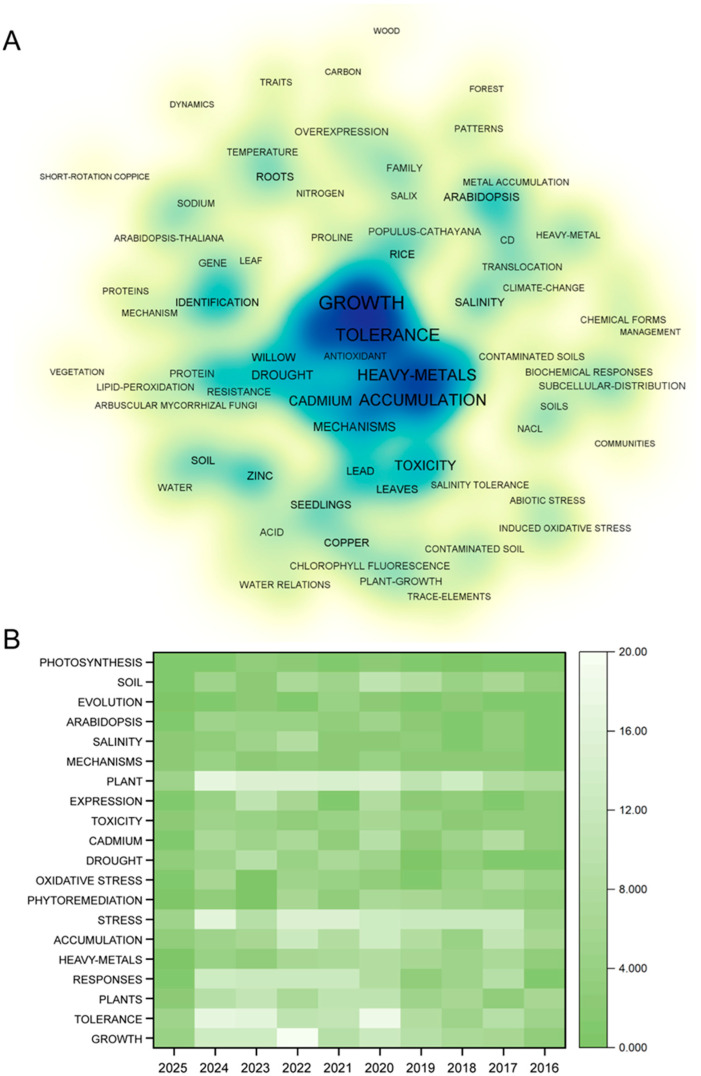
A bibliometric analysis of research on the responses of *Salix* species to abiotic stresses based on the Web of Science Core Collection database. (**A**) Density visualization map of the bibliometric analysis on the responses of Salix species to abiotic stresses, where the color gradient from white to blue indicates a progressive increase in item density. (**B**) Heatmap of bibliometric analysis, the number of documents with the keywords “Salix”, “Abiotic Stresses”, “Cold”, “Heat”, “Drought”, “Heavy Metal”, “Salt”, “Strong Light”, “Waterlogging”, and “Mechanical Damage”.

For the heatmap of the bibliometric analysis, we selected the top 20 keywords by frequency and retrieved literature data for the past ten years. Origin 2025 (version 10.200196) was then used to create the heatmap (Figure 1).

The analysis results showed that in recent years, studies focusing on *Salix* species and their roles in adapting to environmental stresses have increased significantly. According to the results of the network map of the bibliometric analysis, research on the response of *Salix* species to heavy metals is particularly abundant, reflecting the substantial impact of heavy metals, especially cadmium, on the growth and development of *Salix* species. Other stress factors, such as drought, water availability, and NaCl, were also frequently investigated. Most studies have focused on the effects of environmental stress on the growth and development of *Salix* species, whereas molecular mechanisms underlying *Salix* responses to environmental stress remain less explored. The heatmap of the bibliometric analysis yielded similar conclusions to those of the network map, further indicating that research on the responses of *Salix* species to environmental stresses is gaining increasing attention. However, most studies have concentrated on the effects of environmental stresses on plant growth and development, and there is still ample room to advance research on the molecular mechanisms involved.

## 2. Abiotic Stress Responses in Salix Species: Physiological, Morphological, and Ecological Perspectives

*Salix* species inhabit ecologically diverse environments, ranging from alpine zones and arid regions to floodplains and saline–alkaline soils. This wide distribution exposes them to various abiotic stressors, either individually or in combination, with substantial spatiotemporal variability. To cope with these dynamic environmental challenges, willows have developed integrated adaptive mechanisms involving physiological adjustments, morphological plasticity, and ecological niche specialization. A comprehensive analysis of stress types, their ecological contexts, and genus-specific responses is essential for understanding the environmental adaptability and physiological ecology of *Salix*.

### 2.1. Multifaceted Impacts of Drought Stress on Salix Species

Drought exerts widespread and profound effects on *Salix* species, disrupting physiological metabolism, inhibiting growth and reproduction, and weakening ecological adaptability [8,9]. Physiologically, drought induces stomatal closure to limit water loss, but this also suppresses photosynthesis and reduces carbon assimilation [10]. Growth is markedly inhibited, with restricted root development and reduced aboveground biomass, resulting in stunted plants and leaf curling or premature abscission [3,11]. Reproductive processes are similarly impaired: drought reduces floral bud differentiation, pollen viability, seed yield, and germination rate, and also decreases the success of vegetative propagation such as cuttings and root suckers, thus threatening population regeneration [10]. Ecologically, drought drives hydrophilic *Salix* species to retreat to moist habitats, alters community structure, and facilitates replacement by more xerophytic species [12]. Moreover, drought compromises stress resistance, increasing susceptibility to pests and pathogens [13].

### 2.2. Physiological and Ecological Consequences of Salt Stress in Salix Species

Salt stress imposes multifaceted challenges on *Salix* species, primarily through osmotic imbalance, ion toxicity, and oxidative stress. High salinity impairs water uptake, inducing osmotic stress that manifests as leaf wilting, chlorosis, and necrosis [14]. Excessive accumulation of Na^+^ and Cl^−^ disrupts cell membrane integrity [15], inhibits photosynthetic enzyme activity, reduces chlorophyll content, and ultimately diminishes photosynthetic efficiency [14]. Salinity also interferes with the uptake of essential nutrients such as K^+^ and Ca^2+^, leading to metabolic disturbances and growth inhibition [16]. Prolonged exposure severely restricts root development and biomass accumulation, reducing the plant’s capacity for water and nutrient absorption [17]. Reproductive processes are also compromised, with declines in seed germination and seedling survival, ultimately limiting natural population regeneration [18]. Although salt tolerance varies among *Salix* genotypes, salinity broadly constrains their distribution and ecological functionality [19].

### 2.3. Physiological and Reproductive Impacts of Temperature Stress on Salix Species

*Salix* species are highly sensitive to temperature extremes, with both heat and cold stress significantly affecting their physiological metabolism, reproductive success, and ecological adaptability.

High temperature stress compromises cellular homeostasis by disrupting membrane integrity, increasing permeability, and enhancing ion leakage, ultimately impairing water balance [20]. Elevated temperatures denature proteins and reduce the activity of key enzymes such as ribulose-1,5-bisphosphate carboxylase/oxygenase (Rubisco), leading to diminished photosynthetic efficiency. Heat stress also damages chloroplast ultrastructure, accelerates chlorophyll degradation, reduces the stability of photosystem II (PSII), and intensifies photoinhibition. Enhanced respiration rates further deplete carbon reserves and energy [21]. Morphologically, symptoms include leaf scorching, curling, premature senescence and abscission, epidermal sunburn cracking, suppressed apical meristem activity, stunted growth, and reduced biomass accumulation. Reproductively, heat stress decreases pollen viability and stigma receptivity, impairs fertilization, and increases seed abortion rates. Root development in seedlings is also inhibited, weakening subsequent stress resistance [21].

Low temperature stress alters membrane fluidity by inducing a phase shift from the liquid-crystalline to the gel state, increasing permeability and ion leakage, and disrupting intracellular ionic balance. Extracellular ice formation reduces water potential, causing cellular dehydration, while prolonged freezing may lead to intracellular ice crystal formation, resulting in membrane rupture and cell death. Cold stress also inhibits photosynthetic electron transport, reduces chlorophyll synthesis, and lowers light energy utilization [22].

### 2.4. Toxicological and Ecological Effects of Heavy Metal Stress on Salix Species

Heavy metal contamination imposes both toxicological and ecological burdens on *Salix* species [23]. High concentrations of metals such as cadmium (Cd) and copper (Cu) inhibit cell division in root elongation zones, damage the ultrastructure of root tip cells, and cause root browning and atrophy, thereby impairing water and nutrient uptake [24]. At the physiological level, heavy metals disrupt chlorophyll biosynthesis, leading to chlorosis, and displace essential metal cofactors such as Mg^2+^ and Fe^2+^ from enzymatic active sites, impairing the photosynthetic electron transport chain and reducing carbon assimilation [25]. Although some *Salix* species exhibit tolerance mechanisms—such as cell wall immobilization and vacuolar compartmentalization—chronic exposure to heavy metals results in stunted growth, as reflected by reduced plant height and lower annual ring density [26]. Ecologically, heavy metal stress alters the composition of root exudates, which in turn reshapes the diversity and function of rhizosphere microbial communities. This disruption compromises the phytoremediation capacity of willows and their ecological role as pioneer species in polluted environments [27,28].

### 2.5. Metabolic and Morphological Adaptations of Salix Species to Flooding Stress

Flooding stress in *Salix* species triggers a cascade of responses, including hypoxia-induced metabolic reprogramming and subsequent morphological adaptations. In hypoxic conditions, mitochondrial aerobic respiration in roots is inhibited, reducing ATP production. Consequently, plants shift to anaerobic pathways—mainly ethanol and lactic acid fermentation—to meet energy demands. However, these pathways lead to the accumulation of toxic intermediates such as acetaldehyde and lactate [29]. Physiologically, flooding induces prolonged stomatal closure, decreasing photosynthetic rates. Additionally, root hypoxia reduces nitrate reductase activity, impairing nitrogen assimilation, which results in chlorosis in older leaves and thinning of newly developed ones [30]. Extended submergence also triggers programmed cell death in cortical cells, promoting the formation of aerenchyma—a specialized tissue that facilitates internal oxygen transport. However, this adaptation weakens the mechanical strength of the root system [31,32]. Morphologically, flooding induces hydrophytic features such as the proliferation of adventitious roots and basal stem swelling. However, it also causes irreversible damage, including primary root decay and malformation of xylem vessels [33].

### 2.6. Photoinhibition and Adaptive Responses of Salix Species to High Light and UV Stress

High light and ultraviolet (UV) stress impose complex and dynamic effects on *Salix* species, characterized by an ongoing interaction between photodamage and photoprotection. Under intense irradiance, the absorbed light energy exceeds the capacity of the photosynthetic apparatus, leading to accelerated degradation of photosystem II (PSII) reaction center proteins, a decrease in maximum photochemical efficiency, and the onset of photoinhibition [34]. Exposure to UV-B significantly impacts root anatomy and stress tolerance. In *S. nigra* cuttings, UV-B treatment increased root porosity (POR) from 25% in the control group to 47%, representing a physiological adaptation that enhances environmental stress tolerance [31]. Moreover, combined UV-B and elevated temperature stress resulted in a 4–5% reduction in bud length in 2010, with the effect being more pronounced in male plants than in females [35]. However, other studies suggest that vegetative bud size is primarily regulated by seasonal temperature, and UV-B-induced changes are generally weaker and transient [35].

### 2.7. Integrated Comparative Analysis of Abiotic Stress Responses in Salix Species

#### 2.7.1. Common Physiological Adaptations

The diverse abiotic stressors faced by *Salix* species—including drought, salinity, temperature extremes, heavy metals, flooding, and high light/UV—elicit both shared and stress-specific adaptive mechanisms. Common physiological responses across these stressors involve the accumulation of reactive oxygen species (ROS) and subsequent oxidative damage, which plants mitigate through antioxidant systems. Photosynthetic impairment is another frequent consequence, arising from stomatal closure under drought or chloroplast dysfunction under multiple stresses. At the morphological level, most stressors suppress root development, reduce overall biomass, and induce leaf abnormalities such as curling, chlorosis, or premature abscission. Reproductive success is consistently vulnerable, with stressors often reducing pollen viability, seed yield, and seedling establishment, thereby threatening population regeneration.

#### 2.7.2. Unique Stress-Specific Mechanisms

Despite these commonalities, each stressor triggers distinct adaptive strategies. Drought and salt stress both require osmotic adjustment, but salinity uniquely demands ion homeostasis mechanisms to exclude toxic Na^+^ and Cl^−^. Temperature extremes disrupt cellular stability in opposing ways—cold stress alters membrane fluidity, while heat stress causes protein denaturation and exacerbates photoinhibition. Heavy metals induce specialized detoxification pathways, such as cell wall immobilization and vacuolar sequestration, whereas flooding prompts metabolic shifts toward anaerobic respiration and morphological adaptations like aerenchyma formation. High light and UV stress, meanwhile, elicit photoprotective responses, including increased root porosity, but exhibit gender-specific effects on reproductive structures, highlighting the complexity of stress interactions.

#### 2.7.3. Ecological Implications and Habitat Shifts

Ecologically, these responses influence habitat distribution and community dynamics. Drought and salinity often push *Salix* species toward moist or less saline niches, facilitating replacement by more xerophytic or halophytic competitors. In contrast, flooding and heavy metal tolerance reinforce *Salix*’s role as a pioneer species, though chronic stress can impair its phytoremediation capacity. The integration of these physiological, morphological, and ecological strategies underscores *Salix*’s remarkable plasticity, enabling it to navigate trade-offs between stress survival and long-term fitness in unpredictable environments.

Table 1 summarizes the physiological and molecular responses of Salix species to major abiotic stresses, highlighting shared and stress-specific mechanisms that underpin their adaptability.

**Figure 2:**
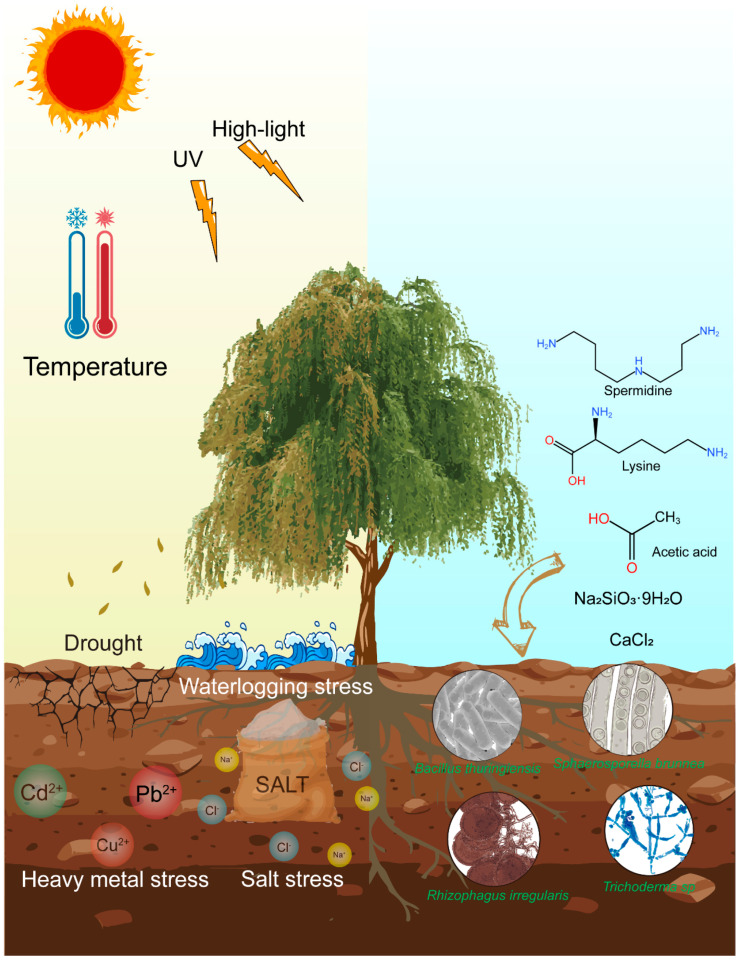
Impacts of multiple abiotic stressors on *Salix* growth dynamics, with synergistic mitigation by exogenous additives and rhizosphere microorganisms.

Figure 2 is a conceptual model illustrating the interactions between multiple abiotic stressors (e.g., drought, salinity, heavy metals) and their effects on *Salix* growth, including reduced biomass, impaired photosynthesis, and oxidative damage. The diagram also shows the mitigating roles of exogenous additives (e.g., lysine, silicon, spermidine) and rhizosphere microorganisms, which enhance antioxidant defenses and osmotic adjustment. This figure is based on a synthesis of the published literature rather than direct experimental data.

## 3. Abiotic Stress Response Mechanisms in Salix: Osmotic Regulation, ROS Signaling, and Hormonal Control

Physiological and biochemical responses form the first line of defense for *Salix* species in response to abiotic stress. These mechanisms enable plants to rapidly adjust their internal environment, maintaining metabolic processes and minimizing cellular damage. Osmotic regulation and antioxidant defense systems act synergistically to alleviate cellular damage caused by stressors such as drought and salinity. Additionally, the regulation of plant hormones plays a crucial role in promoting adaptive responses at the cellular level. Through these integrated mechanisms, willows are able to sustain growth and reproductive capacity under a variety of adverse environmental conditions, demonstrating their remarkable ecological adaptability.

### 3.1. Osmotic Adjustment: Solute Accumulation and Membrane Stability

Osmotic adjustment, a crucial physiological adaptation mechanism, enables plants to maintain turgor pressure and water uptake under abiotic stress through active solute accumulation to reduce cellular osmotic potential [36]. In *Salix* spp., this process is central to their ecological adaptability, facilitating survival across diverse habitats from arid deserts to riparian wetlands [6]. P The osmotic regulation in *Salix* spp. involves coordinated accumulation of multiple solutes under drought and salinity stress, including proline, sucrose, trehalose [22], and inorganic ions (K^+^, Ca^2+^, Na^+^) [37]. Beyond osmotic balance, these compounds stabilize macromolecular structures, scavenge reactive oxygen species (ROS), and maintain membrane integrity. In *S. daphnoides* Vill. and *S. purpurea* L., drought reduced shoot biomass but increased bark proline content. Phenolic compound responses varied by chemical class, species, and stress duration: salicylates remained stable, whereas flavonoids, phenolic acid derivatives, and salireposide exhibited dynamic accumulation patterns under drought [38]. Metabolomic studies in *S. sinopurpurea* and *S. suchowensis* identified 67 and 64 drought-responsive metabolites, respectively. These metabolites functioned as compatible solutes, energy reserves, and antioxidants. Carbohydrate, amino acid, and lipid metabolism were pivotal for drought adaptation, with *S. sinopurpurea* accumulating aspartate, glutamate, serine, threonine, and sedoheptulose—potential contributors to its enhanced drought tolerance. Conversely, *S. suchowensis* exhibited drought sensitivity linked to suppressed phenylalanine and phytosterol biosynthesis [8]. Under salt stress, osmotic regulation remains critical: salinity decreased chlorophyll, carotenoids, and relative water content while elevating proline, soluble sugars, soluble proteins, and Na^+^/K^+^ ratios across tested clones [39].

### 3.2. ROS Signaling and Antioxidant Defense Networks

Studies on abiotic stress responses in *Salix* spp. have demonstrated the central role of reactive oxygen species (ROS) in stress signaling and damage mitigation. Under stress conditions, upregulated genes enhance antioxidant enzyme activities (e.g., superoxide dismutase [SOD] and catalase [CAT]), facilitating ROS detoxification and improving stress tolerance [40]. While ROS-mediated systemic signaling remains uncharacterized in *Salix* spp., evidence from other plants indicates ROS function as dual agents—both damaging molecules and key signaling mediators that regulate gene expression and coordinate with Ca^2+^ to propagate ROS waves for inter-tissue stress signaling [41].

Recent advances elucidate the antioxidant and ROS-signaling networks in *Salix* spp. For example, in *S. matsudana* Koidz., exogenous hydrogen sulfide (H_2_S) and methylglyoxal (MG) alleviated cadmium-induced oxidative stress by regulating glutathione metabolism [42]. Multiple abiotic stressors (flooding, salinity, drought, low temperature) trigger ROS responses in *S. matsudana*, activating antioxidant enzymes and stress-responsive genes [43]. Beyond enzymatic systems, metabolites also mediate ROS signaling under abiotic stress. In *S. matsudana* subjected to combined cadmium and drought stress, excessive ROS accumulation severely inhibited seedling growth (root length and plant height). Salicylic acid (SA) treatment, however, elevated proline biosynthesis via enhanced ornithine aminotransferase (OAT) activity and suppressed proline dehydrogenase (ProDH), while proline exerted multifunctional protective roles in SA-mediated stress adaptation [44].

### 3.3. Regulation of Abiotic Stress Responses in Salix by Plant Hormones

Plant hormones play a critical role in regulating the response of willow species (*Salix* spp.) to abiotic stresses such as drought, salinity, and heavy metal toxicity, coordinating physiological and molecular mechanisms to enhance stress tolerance. Among these hormones, abscisic acid (ABA) is widely recognized as a key signaling molecule in stress responses. Under drought or osmotic stress, ABA rapidly accumulates in leaves, roots, and root tips, contributing to reduced water loss through stomatal closure. For example, significant increases in ABA levels were observed in detached leaves and roots of *S. dasyclados* following dehydration, with higher accumulation in younger tissues compared to mature ones, resulting in rapid stomatal closure and delayed reopening during recovery [45]. In *S. viminalis*, transient elevation of ABA in leaves and apical tissues was associated with a decline in the apical growth rate and bud senescence, coinciding with decreased water potential. Exogenous ABA application suppressed growth and increased stomatal resistance, while gibberellic acid (GA_3_) treatment reversed these inhibitory effects [46]. In *S. pentandra* seedlings, exogenous ABA significantly increased stomatal resistance and reduced transpiration and growth rates, although it failed to induce growth cessation or bud dormancy under a 24 h photoperiod, suggesting that ABA alone is insufficient to trigger photoperiod-mediated dormancy induction [47]. Under combined drought and nitrogen deposition, *S. rehderiana* exhibited altered foliar ABA levels linked to modified flavonoid biosynthesis pathways, ultimately affecting growth dynamics and drought resilience [48].

In addition to ABA, other hormones have also been implicated in willow responses to abiotic stresses. Under arsenic stress in the roots of *S. purpurea*, transcripts encoding ACC synthase, the enzyme responsible for ethylene precursor biosynthesis, were upregulated nearly eightfold, along with the ethylene-insensitive factor (EIN), suggesting activation of ethylene biosynthesis and signaling under metal stress. In contrast, transcripts of ACC oxidases (ACOs) and ethylene response factors (ERFs) were downregulated in stems and leaves, indicating organ-specific regulation of ethylene signaling [49]. In a transcriptomic study of shrub willows under salt stress, the salt-tolerant genotype JW2372 exhibited more differentially expressed genes involved in ethylene signaling compared to the salt-sensitive JW9-6, further supporting ethylene’s role as a key hormone in salt stress adaptation [50].

Salicylic acid (SA) also plays a significant role in chemical stress responses in willows. In *S. matsudana* seedlings, exogenous SA treatment significantly enhanced tolerance to 2,4-dinitrophenol (2,4-DNP), as evidenced by improved photosynthetic efficiency, increased antioxidant enzyme activity, and reduced toxicity, suggesting SA as a regulator of chemical stress resistance [51]. Jasmonic acid (JA) signaling has been implicated in willow responses to drought stress [3]. Comprehensive transcriptomic analyses have revealed that willow responses to salt stress involve crosstalk among multiple hormonal signaling networks, including auxin and cytokinin. However, the specific roles of these hormones in *Salix* species remain insufficiently characterized and require further investigation [50].

In summary, osmotic regulation, ROS signaling, and hormonal pathways constitute a highly integrated and conserved defense system against diverse abiotic stressors in Salix species. Both drought and salinity trigger osmotic adjustments through the accumulation of proline, soluble sugars, and inorganic ions, which contribute to turgor maintenance and cellular protection. ROS production is a universal response; however, the magnitude and composition of antioxidant systems differ by stressor: drought and heat stress commonly activate superoxide dismutase (SOD) and catalase (CAT), while heavy metal stress additionally requires metal-chelating pathways. Hormonal regulation shows extensive cross-talk among ABA, ethylene, and SA pathways, with ABA predominating under drought, salinity, and heavy metal stress, while ethylene and SA display more stressor-specific roles. Overall, as the first line of defense in environmental perception, willows inhabiting harsh environments have developed diverse physiological and biochemical strategies to mitigate abiotic stresses. Over long-term evolution, these species have also evolved stable and enduring molecular regulatory mechanisms. These mechanisms are closely linked to, yet distinct from, the physiological and biochemical responses: they enable rapid responses to transient environmental fluctuations while contributing to long-term adaptation from an evolutionary perspective.

## 4. Molecular Mechanisms of Salix Resistance to Abiotic Stress

**Figure 3:**
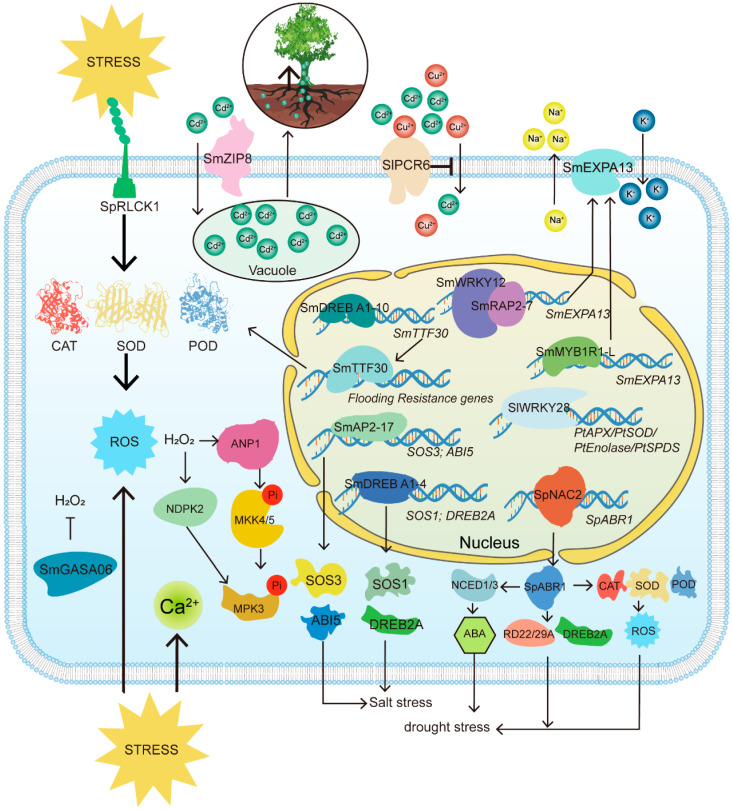
Molecular mechanisms underlying the responses of *Salix* species to abiotic stresses. This figure integrates transcriptomic and proteomic data from published studies to present a hypothetical signaling network involving transcription factors, ROS signaling, and hormone crosstalk. The diagram includes key components (e.g., WRKY, MYB, AP2/ERF transcription factors; ROS; ABA hormones) and their interactions in stress responses. Arrows indicate proposed regulatory relationships based on gene expression patterns and functional studies.

In the preceding section, we examined in detail the mechanisms through which willows respond to abiotic stress, emphasizing osmotic regulation, reactive oxygen species (ROS) signaling, and hormonal control. However, the molecular mechanisms underlying willow resistance to abiotic stress offer considerable research potential beyond these well-established response pathways. Investigating specific molecular pathways and the roles of key genes will provide deeper insights into how willows fine-tune their responses to various stressors. These mechanisms not only involve traditional antioxidant systems but also include processes such as metal ion transport and the regulation of drought-related genes. Such studies are essential for advancing our understanding of willow stress resistance and for improving phytoremediation efforts using these species (Figure 3).

### 4.1. Perception and Signaling Mechanisms of Abiotic Stress in Salix spp.

The plasma membrane is the primary site where *Salix* spp. perceive abiotic stresses. Receptor-like kinases (RLKs) and receptor-like cytoplasmic kinases (RLCKs) on the cell membrane can directly or indirectly detect changes in salinity, osmotic pressure, and other environmental factors, thereby triggering downstream signaling cascades [52]. Although *Salix*-specific RLKs have been scarcely studied, the RLCK family member SpRLCK1 from *S. psammophila* provides valuable insights. SpRLCK1 is a cytoplasm-localized RLCK VIIa protein with high expression in roots and is induced by drought and salt stress. Overexpression of this gene in *Arabidopsis* enhanced ROS-scavenging enzyme activity, thereby improving stress tolerance [53].

Abiotic stresses often induce a transient increase in intracellular calcium ion (Ca^2+^) concentration, which activates calcium-dependent protein kinases (CDPKs) and the calcineurin B-like (CBL)/CBL-interacting protein kinase (CIPK) signaling pathway. External stimuli such as phytohormones, gravity, light, temperature fluctuations, drought, hypoxia, salinity, mechanical injury, and pathogens rapidly elevate cytosolic Ca^2+^ levels. These changes are decoded by downstream effectors to trigger specific biological responses [54]. In the salt-stressed root transcriptome of *S. matsudana*, *CBL* (*SOS3*), *CIPK* (*SOS2*), and the Na^+^/H^+^ antiporter *SOS1* were identified as key differentially expressed genes (DEGs), indicating that the Ca^2+^–CBL/CIPK module plays a central role in salt signal transduction [55].

In response to stress, *Salix* cells also significantly increase the production of ROS. In *S. matsudana*, homologs of NADPH oxidase (RBOH), such as AtRBOHF/D, are upregulated under salt stress, promoting ROS accumulation [56]. ROS act as both damaging byproducts and signaling molecules, mediating kinase activation and antioxidant gene expression. Elevated Ca^2+^ and ROS levels facilitate intracellular signal transduction. Studies have shown that hydrogen peroxide (H_2_O_2_) activates the mitogen-activated protein kinase (MAPK) cascade through phosphorylation of MKK4/5 via upstream activation of ANP1, which subsequently activates MPK3. H_2_O_2_ can also directly activate MPK3 by inducing nucleoside diphosphate kinase 2 (NDPK2) [57]. Moreover, transcriptome analysis of the hybrid *S. matsudana* × *S. alba* under combined salt and flooding stress revealed enrichment of Ca^2+^ signaling, kinase activity, and MAPK pathways. Genes in the MAPK pathway were shown to be essential in mediating responses to salt-flood stress, as stress-induced ROS imbalance rapidly activated the MAPK cascade [58].

### 4.2. Transcription Factors in Salix: Regulatory Modules and Functional Evidence

Upon perceiving stress signals, willow (*Salix* spp.) cells activate a series of regulatory mechanisms, among which transcriptional regulation plays a central role. Transcription factors (TFs) are essential in modulating the expression of stress-responsive genes.

WRKY transcription factors are key regulators of abiotic stress responses. In *S. matsudana*, researchers found that the drought-inducible gene *SmWRKY12* was upregulated in the roots of the drought-tolerant cultivar 9901 under stress conditions, as determined by qPCR analysis. Overexpression of *SmWRKY12* in the callus tissue of *S. matsudana* significantly enhanced drought tolerance. Y1H and dual-luciferase reporter assays demonstrated that *SmWRKY12* activates the *SmEXPA13* promoter and physically interacts with *SmRAP2-7*. Their interaction further strengthened *SmWRKY12*-mediated activation of *SmEXPA13*, which encodes a cell wall-loosening expansin protein. Transgenic expression of *SmEXPA13* improved drought tolerance in both *S. matsudana* and *Nicotiana benthamiana*, highlighting a novel regulatory module involving *SmWRKY12*, *SmRAP2-7*, and *SmEXPA13* [59]. In salt stress studies, the transcription factor *SmMYB1R1-L* was identified in *S. matsudana* as a positive regulator. Overexpression of *SmMYB1R1-L* in willow calli enhanced physiological responses to salt stress, partly through upregulation of *SmEXPA13* [60].

Homeodomain-leucine zipper (HD-Zip) transcription factors are plant-specific and play crucial roles in stress responses. In *S. suchowensis*, 55 *HD-Zip* genes (*SsHD-Zips*) were identified. GO annotation and promoter analysis indicated potential roles in multiple stress responses, while qPCR showed variable expression patterns under salt, PEG, and heat treatments. Co-expression network analysis suggested synergistic interactions among *SsHD-Zip I* genes under stress. Notably, *SsHox34*, *SsHox36*, and *SsHox51* were prominently involved, although downstream targets remain unidentified [61].

Transcriptome profiling of two *Salix* genotypes (salt-sensitive JW9-6 and salt-tolerant JW2372) under salt stress revealed pronounced differential expression of ERF, MYB, NAC, and WRKY TFs in JW9-6, but lacked functional validation [50].

AP2/ERF transcription factors are critical in abiotic stress responses. Using an HMM profile search in the *Salix matsudana* protein database, a total of 364 *AP2/ERF* genes were identified and categorized. RNA-seq data suggested their involvement in salt stress adaptation [62]. A subsequent study isolated *SmAP2-17*, a salt-inducible gene. Overexpression of *SmAP2-17* in *Arabidopsis* increased salt sensitivity, possibly because this gene is a target of salt stress-induced microRNAs. Interestingly, when an optimized codon version of *SmAP2-17* with a modified binding site was introduced into *Arabidopsis*, the transgenic plants exhibited significantly enhanced salt tolerance. A dual-luciferase reporter assay further demonstrated that SmAP2-17 enhances stress tolerance by upregulating *SOS3* and *ABI5* [7].

In *S. suchowensis*, 27 heat shock transcription factor (Hsf) genes (*SsuHsfs*) were identified through whole-genome sequencing and were found to be associated with abiotic stress responses. The expression profiles of these 27 *SsuHsfs* under various tissues and stress conditions (high temperature, drought, salt, and ABA treatment) were analyzed using RT-PCR. The results indicated that *SsuHsfs* are involved in abiotic stress responses. Hsfs regulate heat shock proteins (Hsps), which are crucial for protein stability under stress [63].

In *S. linearistipularis*, the cell number regulator (CNR) family member *SlCNR8* was shown to confer resistance to heavy metals such as Cd, Zn, Cu, Fe, and Mn. Overexpression of *SlCNR8* in poplar seedlings resulted in decreased Cd accumulation and altered expression of metal transporter genes. Subcellular localization studies showed that SlCNR8 is located in both the nucleus and plasma membrane and exhibits transactivation activity in yeast, suggesting its function in regulating metal ion homeostasis [64].

In *S. matsudana*, members of the C-repeat binding factor (CBF) family, including *SmDREB A1-4*, have been identified as homologs of *Arabidopsis AtCBFs* [65]. CBFs are well-characterized transcription factors involved in plant responses to abiotic stresses, particularly cold stress [66]. *SmDREB A1-4* was shown to localize in both the nucleus and cytoplasm, and its promoter contains TC-rich repeats and ABRE motifs associated with NaCl-induced expression. Overexpression of *SmDREB A1-4* in *Arabidopsis* enhanced salt tolerance in transgenic plants, while silencing of this gene via virus-induced gene silencing (VIGS) reduced salt tolerance in willow seedlings. Further studies revealed four putative downstream targets of *SmDREB A1-4*: *RBOHF*, *SOS1*, *ABI5*, and *DREB2A*. Notably, dual-luciferase reporter assays demonstrated that SmDREB A1-4 directly binds to the promoters of *SOS1* and *DREB2A*, indicating that it functions as a transcriptional regulator of these genes in the salt stress response of willow [65].

Despite the well-established roles of transcription factors in plant stress responses, relatively few studies have explored their regulatory roles in willows under abiotic stress. Most existing research has relied on transcriptomic data mining. Future studies should focus on elucidating the transcriptional regulatory networks that underlie abiotic stress adaptation in *Salix* species.

### 4.3. Other Key Genes Involved in Salix Responses to Abiotic Stress

Genes encoding novel antioxidant components, such as *SmGASA06* in *S. matsudana*, enhance H_2_O_2_ scavenging capacity under stress [67]. For details on classical ROS-scavenging enzymes (e.g., CAT, SOD, APX, POD), see Section 3.2.

Metal transporters critically mediate heavy metal homeostasis. In *S. matsudana*, cadmium (Cd) stress induces *SmZIP8*, a Zrt/Irt-like protein that enhances root-to-shoot Cd translocation via subcellular redistribution (vacuolar sequestration over cell wall binding). Transgenic *SmZIP8* expression reduces photosynthetic toxicity by compartmentalizing Cd into leaf epidermal cells, concurrently improving phytoextraction efficiency (elevated accumulation/translocation factors) and stress tolerance [68]. Tobacco expressing *SmZIP8* showed mitigated Cd cytotoxicity through enhanced mitosis, antioxidant activation, and photosynthetic protection [69].

Phytoextraction is a key method in the phytoremediation of heavy metal-contaminated soils [70]. Identification of metal-tolerant plants and key genes involved in metal ion transport is central to this research. A plant cadmium resistance (PCR) gene, *SlPCR6*, was identified in the roots of *S. linearistipularis*, a species known for its heavy metal tolerance. *SlPCR6* expression was induced by Cd stress and localized to the plasma membrane. Its overexpression in transgenic poplar (84K) decreased Cd and Cu accumulation and enhanced tolerance to both metals, without significantly affecting the expression of other known metal transporter genes. These results suggest that *SlPCR6* may directly mediate Cd and Cu transport, offering a candidate gene for improving phytoextraction efficiency via genetic engineering [71].

Pleiotropic drug resistance (PDR) transporters, part of the ABCG subfamily of ATP-binding cassette (ABC) transporters, have been implicated in heavy metal tolerance. In *S. purpurea*, a total of 21 pleiotropic drug resistance (PDR) transporter genes were identified using the BLASTP algorithm, and their promoters contained cis-elements related to various abiotic stress responses. Under heavy metal stress, *PDR* gene expression varied among tissues and in response to different metals. However, the functional roles of these genes remain to be experimentally validated [72].

In *S. matsudana*, whole-genome data analysis identified 28 members of the *PIN* gene family, encoding proteins that mediate auxin transport. Under 200 mM NaCl treatment, transcriptome analysis showed that the expression levels of *SmPIN1e*, *SmPIN1h*, and *SmPIN3c* were higher in salt-tolerant varieties than in salt-sensitive ones, indicating that these genes respond to salt stress. After 4–12 h of flooding treatment, transcriptome data showed that *SmPIN1c*, *SmPIN2c*, and *SmPIN2d* were significantly upregulated in flooding-tolerant varieties. Following 24–48 h of flooding treatment, *SmPIN3b* and *SmPIN3d* exhibited higher expression levels in flooding-tolerant varieties than in flooding-sensitive ones. Other genes, such as *SmPIN2d*, *SmPIN2c*, and *SmPIN5b*, were found to regulate root development in callus cultures of *S. matsudana* [73].

A study on *S. matsudana* also identified *SmCML56*, a member of the calmodulin-like (CML) gene family. Under 100 mM NaCl treatment, overexpression of *SmCML56* in *Arabidopsis* enhanced salt tolerance. Additionally, after 16 days of treatment with 150 mM NaCl, *S. matsudana* overexpression plants exhibited improved salt tolerance, while VIGS-mediated silencing of *SmCML56* reduced salt tolerance, confirming its functional importance in salt stress resistance [74].

The salt tolerance hub gene *SmERF B1–2* was identified in *S. matsudana*. It contributes to salt stress resistance by regulating Na^+^/K^+^ homeostasis [75].

In *S. linearistipularis*, exposure to 50 mM Na_2_CO_3_ stress triggered proline and soluble sugar accumulation for osmotic adjustment, along with enzymatic and non-enzymatic ROS scavenging. A chloroplast-localized rubredoxin gene, *SlRUB*, was highly expressed under alkali stress and associated with proteins involved in photosynthesis and ROS detoxification. Functional studies in *Arabidopsis* suggest that *SlRUB* is crucial for maintaining photosynthetic performance and oxidative balance under alkali stress [76].

Previously discussed transcription factors *SmWRKY12* [59] and *SmMYB1R1-L* [60] regulate *SmEXPA13*, an expansin gene involved in cell wall loosening. *SmEXPA13* expression is induced by salt stress and helps reduce Na^+^ accumulation while enhancing K^+^ uptake. Similarly, *SmEXPA23* also contributes to salt tolerance by maintaining Na^+^/K^+^ balance [77,78].

In another study, the 4-hydroxy-tetrahydrodipicolinate synthase gene *SlDHDPS* from *S. linearistipularis* was found to alleviate salt stress during seed germination and seedling growth by regulating lysine biosynthesis. Exogenous lysine application significantly improved germination and growth under salt stress, with optimal effects observed at 3 mM [79].

### 4.4. Integrated Signaling Pathways: Hormone-ROS-Genes Crosstalk

The crosstalk between hormone signaling, reactive oxygen species (ROS) homeostasis, and stress-responsive genes plays a pivotal role in abiotic stress adaptation in *Salix* species. In *S. matsudana*, overexpression of the AP2/ERF transcription factor *SmAP2-17* significantly upregulated ABA-responsive genes *ABI5* and *SOS3*, conferring enhanced salt tolerance in transgenic *Arabidopsis thaliana*, highlighting the critical role of ABA signaling components in *Salix* stress adaptation [7]. Similarly, in *S. psammophila*, the transcription factor *SpABR1* (a GRAM domain-containing protein) was identified as a key positive regulator of drought tolerance. *SpABR1* overexpression reduced ROS accumulation and boosted antioxidant enzyme activity by activating ABA biosynthesis and responsive genes, including *DREB2A*, *RD22*, and *RD29*, which were markedly upregulated under osmotic stress [80]. Further mechanistic studies revealed that the NAC family member *SpNAC2* binds to the *SpABR1* promoter, activating its expression. Transgenic *Arabidopsis* overexpressing *SpABR1* exhibited enhanced drought tolerance, increased ABA sensitivity, and elevated antioxidant activity, demonstrating the hierarchical regulation within this pathway [80].

Another NAC family gene, *SpsNAC005* from *S. psammophila*, enhanced salt and drought tolerance when overexpressed in *Populus hopeiensis*. Transgenic plants showed higher SOD and POD activities, increased proline content, and reduced MDA levels, though intriguingly, the expression of *SOS1*, *MPK6*, *HKT1*, *P5CS1*, and *PRODH1* was downregulated, suggesting a complex regulatory network [81]. In *S. linearistipularis*, *SlWRKY28* enhanced alkali-salt tolerance by upregulating antioxidant genes (*PtAPX*, *PtSOD*, *PtEnolase*, and *PtSPDS*) while minimally affecting *PtP5CS* expression, underscoring the specificity of transcriptional regulation in ROS scavenging [82].

Beyond drought and salt stress, *Salix* species employ conserved mechanisms for flooding tolerance. In *S. matsudana* Koidz, 78 Trihelix transcription factors (*SmTTFs*) were identified, with *SmTTF30* rapidly induced by flooding. Overexpression of *SmTTF30* in *Arabidopsis* enhanced flood tolerance, evidenced by higher leaf cell viability, elevated POD activity, and reduced MDA levels under stress [43]. Further analysis revealed that SmDREB A1-10 directly binds to the *SmTTF30* promoter, functioning upstream of it. Silencing SmDREB A1-10 suppressed *SmTTF30* and hypoxia-responsive genes, exacerbating submergence stress [1].

In summary, these integrated signaling pathways demonstrate the complex interplay between hormones, ROS, and transcription factors in *Salix* spp. under abiotic stress. The convergence of these pathways orchestrates gene expression and physiological adjustments that collectively enhance stress adaptation (Table 2).

In addition to transcriptional regulation, emerging evidence suggests that epigenetic regulation, non-coding RNAs, and post-translational modifications play important roles in plant responses to abiotic stresses. Although limited data are currently available in *Salix* spp., studies in other woody plants have highlighted the roles of DNA methylation and histone modifications in modulating stress-responsive gene expression and stress memory. Non-coding RNAs, such as microRNAs (miRNAs) and long non-coding RNAs (lncRNAs), are known to fine-tune the expression of key transcription factors and signaling molecules under stress. Moreover, post-translational modifications, including phosphorylation, ubiquitination, and sumoylation, dynamically regulate the stability, activity, and subcellular localization of stress-responsive proteins, thereby contributing to rapid stress adaptation.

Future studies should integrate multi-omics approaches to systematically investigate these regulatory layers in *Salix* spp., which will help build a more comprehensive model of stress adaptation and provide potential targets for improving stress resilience through breeding or biotechnological approaches.

In conclusion, the molecular mechanisms of Salix responses to abiotic stresses involve both common and stress-specific regulatory modules. Transcription factors such as WRKY, MYB, and AP2/ERF families consistently contribute to stress tolerance by modulating downstream stress-responsive genes. Notably, WRKY factors are broadly involved in drought, salt, and heavy metal stress, while AP2/ERF factors exhibit roles in salinity and cold stress. Shared signal transduction elements, including receptor-like kinases (RLKs), calcium-dependent protein kinases (CDPKs), and ROS-producing NADPH oxidases, are repeatedly recruited under various stress conditions. However, stress-specific differences emerge, such as the prominent role of metallothioneins and metal transporters under heavy metal stress, and the unique induction of aquaporins and cell wall-modifying genes during flooding. Understanding both the shared and unique regulatory mechanisms will be key to breeding Salix cultivars with broad-spectrum stress resilience.

## 5. Abiotic Stress Response Strategies: Male vs. Female Willows

Willows (*Salix* spp.) are dioecious, with sex determination mechanisms varying among species due to differences in sex chromosome evolution and the localization of sex-determining regions (SDRs) [83]. In the ZW system, females are heterogametic (ZW), while males are homogametic (ZZ). In contrast, the XY system involves heterogametic males (XY) and homogametic females (XX) [84]. Some *Salix* species exhibit a transition from XY to ZW systems [85]. Many studies have demonstrated sexually dimorphic responses to abiotic stresses in willows.

### 5.1. Morphological and Physiological Differences

Under drought and high nitrogen deposition, female *S. myrtillacea* cuttings exhibit superior growth and drought resistance compared to males, attributed to enhanced water acquisition, higher water use efficiency (WUE), elevated foliar ABA and IAA levels, and better ROS scavenging capacity [11,48]. In *S. paraplesia*, drought reduced growth and photosynthesis in both sexes; however, females showed higher biomass, chlorophyll content, and antioxidant enzyme activity, indicating superior drought tolerance [86].

Interestingly, in *S. matsudana*, males developed stronger root systems and higher biomass under salinity stress [87]. In *S. viminalis* exposed to cadmium stress, females initially accumulated more H_2_O_2_ and developed larger roots, but over time, males exhibited higher cadmium tolerance with increased antioxidant enzyme activity [88].

### 5.2. Hormonal and Metabolite Profiles

Exogenous acetic acid (AA) enhances drought resistance in *S. rehderiana*, *S. babylonica*, and *S. matsudana*, with females showing greater improvements in root development, osmotic adjustment, antioxidant activity, and photosynthesis [89]. AA also modulates jasmonic acid signaling, activating it in females while suppressing it in males, contributing to sex-specific drought responses [3].

Proteomic and acetylproteomic analyses in *S. myrtillacea* revealed that drought-tolerant females suffered less photosynthetic and oxidative damage, while drought-sensitive males activated acetyl-CoA biosynthesis, fatty acid metabolism, and jasmonic acid pathways [5]. Under salt stress in *S. linearistipularis*, female plants exhibited higher SOD and POD activities, lower H_2_O_2_ content, and enhanced photosynthetic capacity compared to males [79,90]. Females also had lower Na^+^ accumulation in leaves and higher Na^+^ efflux in roots, likely due to higher expression of *SlNHX3*, *5*, *6*, and *7* [79,90].

### 5.3. Transcriptional and Molecular Differences

Transcriptomic analysis of *S. matsudana* under salinity stress showed that both sexes upregulated stress-related genes (e.g., ADH, oxygenase-related genes); however, males additionally upregulated other abiotic stress response genes, while females downregulated nitrogen metabolism genes to mitigate salt damage [87]. Under combined drought and low-temperature stress, *S. myrtillacea* females accumulated more amino acids and sugar alcohols, while males produced more flavonoids, indicating a trade-off between growth and defense strategies [22].

Proteomic studies revealed that exogenous AA regulated acetyl-CoA metabolism differently in males and females, contributing to stress-specific performance [5]. Notably, LysAc (lysine acetylation) modifications also showed sexual dimorphism in histones, transcription factors, and metabolic enzymes, affecting stress-responsive gene expression [5].

Overall, *Salix* species exhibit sexually dimorphic responses to abiotic stresses at morphological, physiological, hormonal, metabolite, and transcriptional levels. These differences highlight evolutionary trade-offs between growth and defense strategies, driven by complex gene regulatory networks and epigenetic modifications. Understanding these mechanisms is essential for developing stress-resilient willow cultivars.

## 6. Exogenous Treatments and Microbial Interactions in Enhancing Salix Resistance to Abiotic Stresses

### 6.1. Exogenous Additives: Amino Acids, Hormones, and Elements

As previously mentioned, lysine plays a crucial role in helping plants resist salt stress. Exogenous lysine supplementation alleviates salt stress phenotypes during germination and seedling development in *Salix* spp. [79]. In a study on *S. matsudana* Koidz., it was found that the application of silicon (Si) reduced cadmium accumulation by inhibiting plant cadmium absorption, while spermidine (Spd) alleviated cadmium toxicity by enhancing antioxidant capacity. This was primarily due to exogenous Si and Spd increasing antioxidant enzyme activity and reducing oxidative damage caused by cadmium [4]. Moreover, exogenous calcium has also been reported to promote the growth of *S. matsudana* seedlings under NaCl stress. This positive effect was linked to enhanced activities of antioxidant enzymes such as catalase (CAT), superoxide dismutase (SOD), and peroxidase (POD), as observed in *Ulmus pumila* seedlings under different NaCl concentrations [91].

Furthermore, as previously mentioned, exogenous acetic acid (AA) effectively improves drought resistance in *Salix* spp. Female willows treated with AA exhibited more extensive root systems, stronger root vitality, enhanced osmoregulatory capacity, increased antioxidant activity, and higher photosynthetic rates, along with lower reactive oxygen species (ROS) levels and fewer stomatal closures mediated by abscisic acid. AA treatment also enhanced the jasmonic acid signaling pathway in female willows but suppressed it in males, which resulted in superior drought resistance in females compared to males. Overall, AA application enhanced drought resistance more in female than male willows, further emphasizing the sexual dimorphism under drought stress [3]. This was mainly due to AA irrigation significantly increasing the relative content of amino acid metabolites (e.g., glycyl-l-tyrosine, l-glutamine, and seryl-tryptophan) and decreasing phenylpropanoid metabolite levels (e.g., resveratrol and sinapyl aldehyde) in the soil. The enrichment of nitrogen-fixing bacteria (Azotobacter) and *Pseudomonas* in the rhizosphere was significantly correlated with the accumulation of these metabolites, promoting nitrogen uptake in *Salix* spp. and improving drought stress resistance [89].

### 6.2. Mycorrhizal Symbioses and Phytoremediation Enhancement

In a study of the response of *S. linearistipularis* to saline–alkaline stress, plant growth-promoting rhizobacteria (PGPR) played a crucial role. In a study of the response of *S. linearistipularis* to saline–alkaline stress, plant growth-promoting PGPR played a crucial role. Inoculation with *Trichoderma* sp. strains M4 and M5 increased proline and soluble sugar contents, enhanced SOD, POD, CAT, and APX activities, and reduced lipid peroxidation; strain M4 was more effective. M4 alleviated seedling damage by lowering oxidative stress, boosting organic acid and amino acid metabolism, and activating phenylpropanoid biosynthesis to scavenge reactive oxygen species [92].

From the roots of *S. alba*, *Bacillus thuringiensis* (Accession MW979616) was isolated. Inoculation of wheat (*Triticum aestivum*) seeds with this strain and root powder markedly improved biomass accumulation and antioxidant enzyme activities under cadmium stress [93].

*Salix* spp., being dual mycorrhizal plants, associate with both arbuscular (AM) and ectomycorrhizal (EM) fungi. In *S. miyabeana*, inoculation with the EM fungus *Sphaerosporella brunnea*, the AM fungus *Rhizophagus irregularis*, or both was tested across two growing seasons. *S. brunnea* notably promoted biomass production and phytoextraction of heavy metals—particularly Cd—and contributed to reductions in certain soil metal concentrations. AM or dual inoculation had minimal effects on these parameters [94].

**Figure 4:**
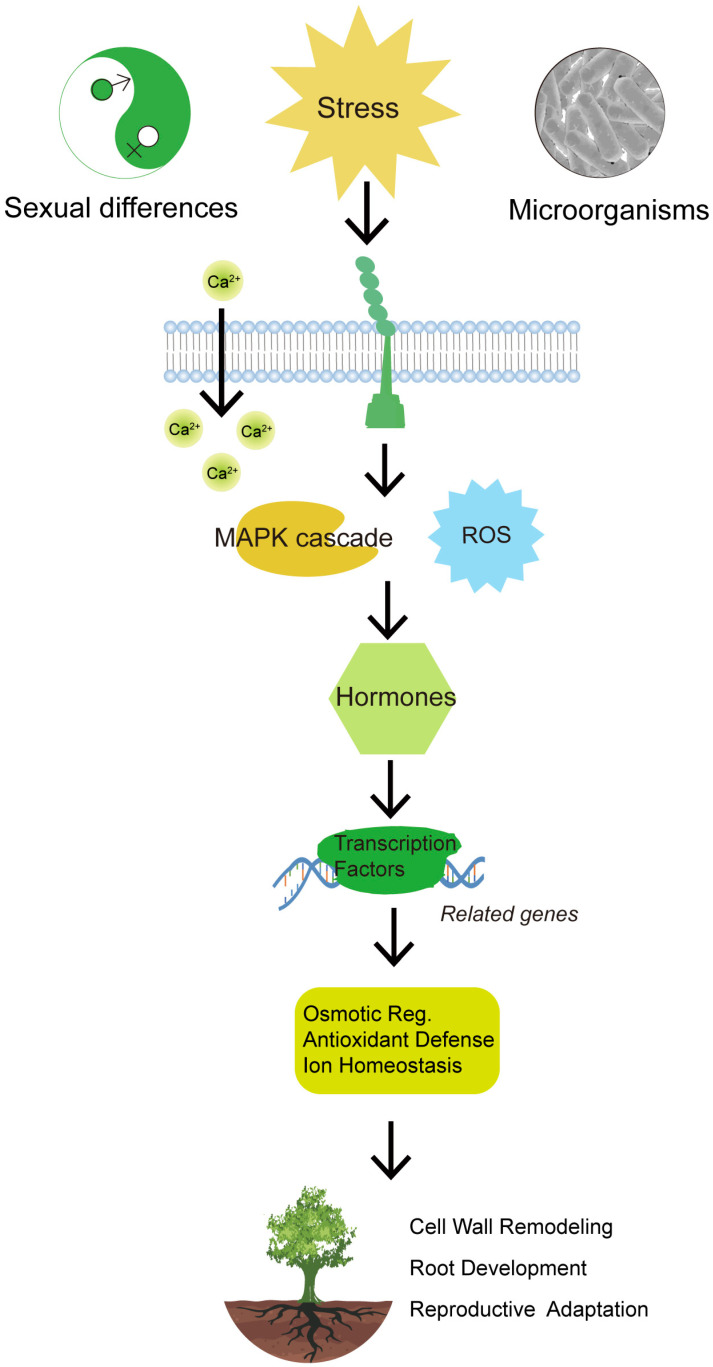
A schematic overview of the core signaling pathways involved in abiotic stress adaptation in *Salix* species.

Figure 4 conceptually illustrates the multifaceted impacts of abiotic stressors on *Salix* growth dynamics, including the interplay between stressors and mitigation strategies by exogenous additives and rhizosphere microorganisms. This conceptual framework helps contextualize the subsequent discussion of physiological and molecular responses.

## 7. Discussion and Future Perspectives

This review provides an integrated perspective on the physiological, molecular, and sex-dimorphic responses of *Salix* species to diverse abiotic stressors. While significant advances have been made in identifying key stress-related pathways, much of the current understanding remains fragmented and largely descriptive. A more critical evaluation reveals that different *Salix* genotypes and species exhibit distinct adaptive strategies under specific stress conditions. For instance, drought-tolerant species such as *S. sinopurpurea* display enhanced osmotic regulation and metabolite accumulation, while *S. suchowensis* shows reduced resilience due to suppression of key metabolic pathways. Under salt stress, *S. matsudana* maintains superior ion homeostasis and water retention, contrasting with salt-sensitive genotypes like *JW9-6*. Similar genotypic variation is observed under heavy metal exposure, where *S. linearistipularis* demonstrates more effective detoxification mechanisms than *S. purpurea*. These differences highlight the necessity of comparative studies across genotypes and species to uncover the genetic basis of stress adaptation and inform targeted breeding efforts.

Moreover, sexual dimorphism introduces another layer of complexity to *Salix* stress responses. Female individuals often exhibit enhanced ROS scavenging, hormonal regulation, and osmotic adjustment under drought, while males may show greater resilience under salinity or flooding through improved root architecture and ion transport. These divergent strategies reflect sex-specific regulatory mechanisms and trade-offs between growth and defense, which remain underexplored in current research. Understanding how male and female *Salix* integrate hormonal signals, epigenetic regulation, and metabolic shifts under stress will be crucial for developing comprehensive stress-resilience models and for sex-informed breeding strategies.

Despite the identification of numerous transcription factors such as WRKY, MYB, NAC, and AP2/ERF that modulate stress responses, functional characterization in *Salix* lags behind. Much of the existing evidence is derived from transcriptomic profiling or heterologous expression in *Arabidopsis*, which cannot fully capture the physiological and developmental context of woody perennials. The lack of efficient transformation systems in *Salix* poses a significant bottleneck for functional genomics research. Future studies must prioritize the development of species-specific gene editing and transformation platforms to enable in situ validation of candidate genes. This will be essential for moving from gene discovery to practical applications in breeding and ecological restoration.

Furthermore, most current studies focus on individual stressors, whereas plants in natural environments are frequently exposed to multiple simultaneous stresses. Drought, salinity, heat, and heavy metals often co-occur, resulting in complex interactions that cannot be inferred from single-stress models. The crosstalk among hormone signaling pathways, ROS homeostasis, and transcriptional regulation under combined stress conditions remains poorly understood in *Salix*. Integrative multi-omics approaches—including transcriptomics, proteomics, metabolomics, and epigenomics—particularly at the single-cell or spatial resolution level, are needed to dissect these complex networks and reveal how plants prioritize and coordinate responses under multifactorial stress.

In addition to intrinsic genetic mechanisms, increasing attention should be given to the role of rhizosphere microbiomes in enhancing stress tolerance. Symbiotic interactions with beneficial microbes, including nitrogen-fixing bacteria, mycorrhizal fungi, and plant growth-promoting rhizobacteria (PGPR), have been shown to improve nutrient acquisition, modulate hormonal balance, and reduce oxidative stress in *Salix*. Microbiome engineering, when integrated with host genetic improvement, holds great promise for building resilient plant-microbe systems adapted to adverse environments.

Practical applications such as phytoremediation, ecological restoration, and biomass production will greatly benefit from these mechanistic insights. Genomic-assisted breeding, including genome-wide association studies (GWAS), genomic selection, and pan-genome analysis, should be implemented to accelerate the development of stress-resilient *Salix* cultivars. Future research should also incorporate field-based phenotyping under natural or simulated multi-stress conditions to ensure translational relevance.

In conclusion, while significant progress has been made in elucidating the abiotic stress responses of *Salix* species, further depth is needed through cross-species comparisons, functional validation, and systems-level analysis. A concerted effort to integrate molecular biology, physiology, microbial ecology, and breeding science will be essential for translating laboratory findings into practical outcomes. Advancing this field will not only contribute to fundamental plant science but also support the deployment of *Salix* as a keystone genus for ecological and environmental resilience in a changing world.

## Figures and Tables

**Table 1:** Physiological and molecular responses of *Salix* to major abiotic stresses.

Stress Type	Species	Physiological Responses	Molecular Responses
Drought	*S. sinopurpurea*, *S. suchowensis*	Stomatal closure, reduced photosynthesis, proline accumulation	Upregulation of WRKY, NAC TFs, ABA signaling
Salt	*S. matsudana*, *S. linearistipularis*	Osmotic imbalance, Na^+^/K^+^ ratio increase	Ca^2+^-CBL/CIPK pathway, AP2/ERF, MYB TFs
Heavy Metal	*S. linearistipularis*, *S. purpurea*	Reduced biomass, chlorosis, ROS surge	Metal transporters, ROS scavenging
Flooding	*S. matsudana*, *S. viminalis*	Hypoxia, aerenchyma formation, stomatal closure	Trihelix TFs, hypoxia-responsive genes
Temperature	*S. myrtillacea*, *S. alba*	Membrane damage, reduced photosynthesis	CBF, Hsf TFs, ROS signaling
High Light/UV	*S. nigra*, *S. myrsinifolia*	Photoinhibition, increased root porosity	Photoprotective genes

**Table 2:** Summary of key transcription factors, stress type, downstream targets, and species.

TF Family	Gene Name	Stress Type	Species	Validation Evidence	Downstream Targets
WRKY	*SmWRKY12*	Drought	*S. matsudana*	Overexpression (callus)	SmEXPA13 (cell wall loosening)
WRKY	*SlWRKY28*	Salt	*S. linearistipularis*	-	Antioxidant genes
	*SmRAP2-7*	Drought	*S. matsudana*	-	SmEXPA13 (cell wall loosening)
NAC	*SpNAC2*	Drought/Salt	*S. psammophila*	Overexpression (Arabidopsis)	SpABR1 (ABA signaling, antioxidants)
Trihelix	*SmTTF30*	Flooding	*S. matsudana*	Overexpression (Arabidopsis)	Hypoxia-responsive genes
AP2/ERF	*SmAP2-17*	Salt	*S. matsudana*	Transgenic Arabidopsis	SOS3, ABI5 (salt stress response)
HD-Zip	*SsHox36*	Salt/Heat	*S. suchowensis*	Transcriptome only	Stress-related genes
DREB	*SmDREB A1-10*	Waterlogging	*S. matsudana*	Overexpression Arabidopsis and Salix VIGS	*SmTTF30*
DREB	*SmDREB A1-4*	Salt	*S. matsudana*	Overexpression Arabidopsis and Salix VIGS	*SOS1* and *DREB2A*
MYB	*SmMYB1R1-L*	Salt	*S. matsudana*	Overexpression (callus)	SmEXPA13 (cell wall loosening)
CNR	*SlCNR8*	Heavy Metal	*S. linearistipularis*	Overexpression (poplar)	Metal homeostasis genes
